# Women's breastfeeding experiences following a significant primary postpartum haemorrhage: A multicentre cohort study

**DOI:** 10.1186/1746-4358-5-5

**Published:** 2010-05-27

**Authors:** Jane F Thompson, Laura J Heal, Christine L Roberts, David A Ellwood

**Affiliations:** 1 Women's Hospitals Australasia, Australian Capital Territory, Australia; 2 The Australian National University Medical School, Australian Capital Territory, Australia; 3 Clinical and Population Perinatal Research, Kolling Institute of Medical Research, University of Sydney, New South Wales, Australia; 4 Department of Obstetrics and Gynaecology, The Australian National University Medical School, The Canberra Hospital, Australian Capital Territory, Australia

## Abstract

**Background:**

Postpartum haemorrhage (PPH) is a significant and increasing contributor to maternal mortality and morbidity. Following a PPH, women may have difficulties initiating and sustaining breastfeeding, although little has been published on this issue. The aim of this study was to describe breastfeeding experiences in a cohort of women following a significant PPH.

**Methods:**

This is a descriptive study based on quantitative and qualitative data collected via questionnaires completed in the first week postpartum and at two and four months postpartum, by 206 women participating in a multicentre study of women's experiences of a significant primary postpartum haemorrhage (blood loss of 1500 mL or more in the 24 hours following childbirth, and/or a peripartum fall in haemoglobin (Hb) concentration to 7g/dL or less, or of ≥ 4g/dL).

**Results:**

Among women with a significant PPH, 63% fully breastfed their babies from birth, whereas 85% said they had hoped to do so (p < 0.001). Only 52% of mothers who intended to either fully or partially breastfeed were able to give their baby the opportunity to suckle within an hour of the birth. Delays were longer in women with greater estimated blood loss and women with the longest delays in breastfeeding were less likely to initiate full breastfeeding. 70% of women with PPH of < 2000 mL were fully breastfeeding in the first postpartum week, whereas less than 50% of those with blood loss ≥ 3000 mL were able to do so. Overall, 58% of women with significant PPH were fully breastfeeding at two and 45% at four months postpartum.

In qualitative data, three major themes were identified: 1) Difficulty initiating or sustaining breastfeeding, 2) Need for education and support; and 3) Emotional sequelae.

**Conclusions:**

Following a significant PPH, women with greater blood loss are less likely to initiate and sustain full breastfeeding and this may be related, in part, to delays in initial contact with their baby as a consequence of the PPH. These findings have implications for postnatal care as these women may require greater support, education and assistance in initiating and sustaining breastfeeding. In particular, enabling the opportunity for the newborn to suckle as soon as is practicable should be encouraged.

## Background

"... ...my dream of breastfeeding has been shattered, due to no energy because of the PPH......."

PPH is a common complication of pregnancy worldwide and a significant contributor to maternal mortality and morbidity [1,2]. Data from Australia, Canada and the United Kingdom indicate rates of PPH and adverse maternal outcomes (including death) attributable to PPH, are increasing [2-5]. However, little information has been published on the short/medium and long term consequences for women who survive a PPH. In particular, the impact of PPH on breastfeeding success has received little attention. Women suffering a PPH may experience a transient hypotensive insult and pituitary ischaemia and/or infarction resulting in inhibition of the hormonal triggering of lactogenesis Stage II by prolactin [6]. In rare cases, women who bleed severely during childbirth may develop Sheehan's syndrome, or ischaemic necrosis of the pituitary gland, in particular of the anterior lobe, secondary to hypoperfusion [7-9]. Failure to lactate or difficulties with lactation, due to absent or deficient prolactin secretion, are common initial symptoms of Sheehan's syndrome [10,11].

In addition, elevated cortisol levels following such a stressful labour and delivery may also adversely affect lactogenesis Stage II [12]. Delayed early contact between mother and baby following a complicated birth with PPH may also impact on a mother's ability to successfully establish and maintain breastfeeding. The only study published to date which specifically examines an association between PPH and breastfeeding is a small case series describing insufficient milk syndrome and failure to thrive in infants associated with maternal PPH [6]. Lack of success in breastfeeding is not benign, and women who are unable to realise their expectation to breastfeed successfully may experience a sense of failure, loss and grief [13].

Early initiation of breastfeeding, within one hour of birth, is recommended by the World Health Organization (WHO) and the United Nations Children's Fund (UNICEF) to stimulate breast milk production, to increase uterine activity (thereby reducing the risk of heavy bleeding and infection), to foster mother-child bonding and increase the duration of breastfeeding [14,15]. Further, exclusive breastfeeding is recommended from birth to six months postpartum [16], and women who report partial breastfeeding (supplementing breast milk with formula) in the first week are more likely to have ceased breastfeeding within six months compared with women who do not supplement with formula in the first week [17]. Australian and New Zealand women have high rates of initiation of at least some breastfeeding in comparison with comparable populations such as in the UK and USA [18-21]. Nonetheless, it is of concern that rising rates of PPH may have a detrimental effect on breastfeeding success in these and other populations.

The overall aim of this study was to determine the short and long term health outcomes for a cohort of Australian and New Zealand women who experienced a significant primary PPH. Here we report the breastfeeding initiation rates and continuation/cessation rates of breastfeeding at two and four months postpartum, as well as on women's accounts of their breastfeeding experiences.

## Methods

The study population included women with a significant PPH defined as: an estimated blood loss of 1500 mL or more in the 24 hours following childbirth, and/or a peripartum fall in haemoglobin (Hb) concentration to 7g/dL or less, and/or a peripartum fall in haemoglobin concentration of ≥ 4g/dL. Women were not included if they were: aged less than 18, did not have sufficient competency in the English language to complete the questionnaires, were unable to complete the questionnaires for other reasons, or if they experienced either a stillbirth or a neonatal death in the index pregnancy.

The study was multi-centred with participants recruited from 17 hospitals with maternity services in Australia and New Zealand between January 2006 and April 2007. Follow-up was completed by September 2007. Hospital staff identified eligible women during their postpartum stay in hospital, provided them with information about the study and then invited them to participate. Once recruited, participants' medical records were reviewed and data were abstracted on obstetric history, pregnancy factors, labour and delivery in the index pregnancy, estimated blood loss in 24 hours postpartum and PPH management. In addition, study participants completed three questionnaires, the first in hospital and the second and third at two and four months postpartum. The latter two were mailed to participants with a pre-paid reply envelope. Non-responders were mailed a reminder card if they did not return their questionnaire within two weeks with a further reminder card or phone call after another two weeks if necessary.

The research was approved by Human Research Ethics Committees for all participating hospitals and written informed consent was obtained from all participants.

### Quantitative data

In the first questionnaire, women provided demographic information and were asked how they had hoped to feed their babies and their current feeding method. In addition, they were asked about the location of their baby in the first hour after the birth and subsequently, and how soon after birth they were given the opportunity to breastfeed. At two and four months postpartum, women were asked about their infant's current feeding method and to rate their baby's health (*1= Poor, 2 = Fair, 3= Good, 4= Excellent*). Breastfeeding definitions were in accordance with WHO definitions viz: *Fully breastfeeding *(exclusive + predominant breastfeeding where the infant receives breast milk, drops or syrups and/or certain liquids); *Complementary feeding *(referred to here as partially breastfeeding - where the infant receives breast milk and also any food or liquid, including non-human milk);* Bottle/formula feeding *(where the infant does not receive any breast milk) [22].

At two and four months postpartum, women were asked to identify their postpartum physical concerns, including breast infection or mastitis, during the preceding two months. Response options for each listed concern were: *'Not a problem', 'A minor problem' *or *'A major problem'*.

### Qualitative data collection

In all three questionnaires, women were invited to provide open ended comments in response to the question: *Is there anything about your labour and birth that is bothering you now*? At two months postpartum two additional questions inviting open-ended responses were also included. The first followed a series of closed response questions about satisfaction with care and information provided in hospital and was worded: *What other information, if any, would you have found helpful? *The second asked: *Are there any other comments you would like to make about your care in hospital and since discharge? *Blank pages were provided at the end of all the questionnaires for women to write any further comments if they wished.

### Sample size and data analyses

A cohort of 200 women was required to give adequate precision (≤ 5%) for the estimate of prevalence of the study outcome measure with the highest expected prevalence (score > 12 on the Edinburgh Postnatal Depression Scale) [23].

Differences between unrelated proportions were tested using Chi Squared tests. McNemar's test was used to determine the significance of the difference between paired proportions. We calculated univariate odds ratios (OR) for exposures of interest to determine their association with not fully breastfeeding (either partially breastfeeding or bottle/formula feeding). Adjusted ORs were calculated using multivariable logistic regression models. Variables were tested for interactions and collinearity. Participants with missing values for any variables of interest were excluded from the multivariable analyses. All tests of statistical significance were carried out at the 5% two sided level. Statistical analyses were conducted using STATA 9.2 for Windows and EpiInfo Version 6 Statcalc module.

For analysis of the qualitative data, women's written comments were transcribed verbatim and analysed for content and inductively coded to identify a thematic framework.

## Results

### Participants

Two hundred and six women consented to participate and completed the first participant questionnaire. The number of women recruited by each hospital varied between 1 (0.5%) and 45 (22%). Of the women who completed the first questionnaire, 171 (83%) also completed the two month and 167 (81%) the four month follow-up questionnaire respectively. 160 (78%) women completed all phases of the study (7 women completed the third but not the second questionnaire). The primary reasons for loss to follow up were failure to return the questionnaire after reminders and inability to contact due to change to unknown address.

Of the 206 women, 182 (88%), met the blood loss eligibility criterion (estimated blood loss 1500 mL or more). The remainder had an estimated blood loss of less than 1500 mL but met the peripartum changes in Hb criteria: 4 women (2%) had a peripartum fall in Hb concentration to 7g/dL, 13 (6%) had a peripartum fall in Hb concentration of ≥ 4g/dL and 7 (3%) met both of the latter two criteria. Overall, estimated blood loss in the first 24 hours postpartum ranged from 300 mL to 8000 mL (median 1800 mL), with 126 women with estimated blood loss < 2000 mL, 56 with 2000-2999 mL and 24 with 3000 mL or more.

### Characteristics of study participants

Of the 206 women, 110 (53%) were primiparous; 196 (95%) women had a singleton birth; the majority 140 (68%) were aged less than 35, with only 2% aged less than 20 and 5% 40 years or older. Overall, 78 (38%) women had a caesarean section, 174 (84%) delivered at term and 19% of babies weighed four kilograms or more.

Following the birth, 62 (30%) participants were admitted to a High Dependency Unit (HDU) or an Intensive Care Unit (ICU). One woman required assisted ventilation in the ICU. Length of stay ranged from one day or less (66%) to five days (1 participant). Reasons for admission to either HDU or ICU were not well reported, but for those 16 women where a reason was recorded all related to complications associated with the PPH.

### Early contact with baby

Data were available for 207 babies of mothers with significant PPH about their location in the first hour after birth. Less than a third (28%) were primarily in their mother's arms. The remainder were either in their father's or another family member's arms (44%) or with hospital staff for routine or special/intensive care (28%). Twins were more likely to be separated from their mothers than singletons (75% versus 22%) primarily because they required special or intensive care. After the first hour after the birth, two thirds (63%) of the babies were able to stay with their mothers all of the time for the rest of their hospital stay. The main reason for separation was because babies required special or intensive care.

### Initiation of breastfeeding

Among women in the cohort, 130 (63%) fully breastfed their babies from birth, whereas 174 (85%) said they had hoped to do so (p < 0.001).

**Figure 1:**
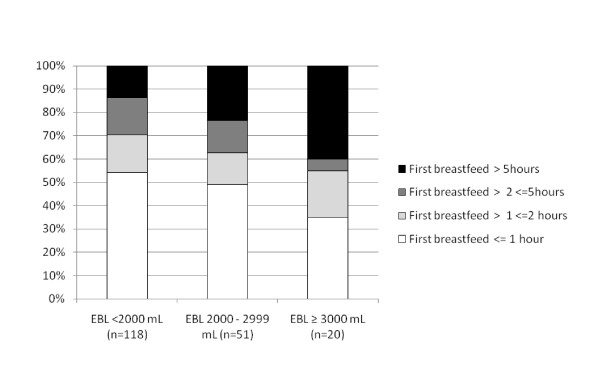
**Timing of first opportunity to suckle among mothers with significant PPH who intended to breastfeed, by estimated blood loss (EBL)**.

**Figure 2:**
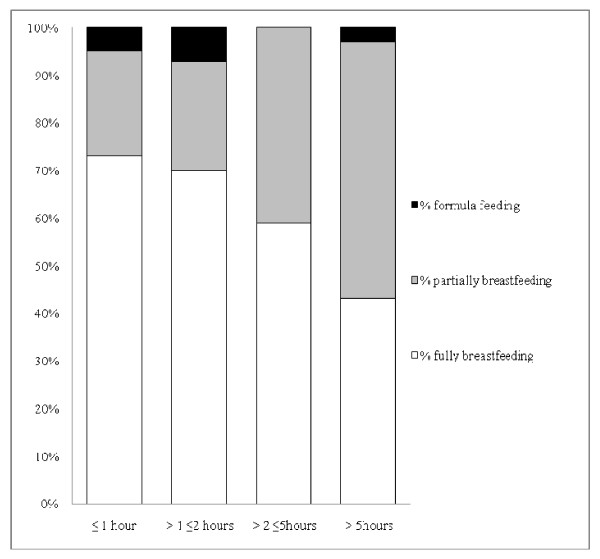
**Breastfeeding status in first postpartum week among mothers with significant PPH who intended to breastfeed, by timing of first opportunity to suckle**.

Just over a half of the mothers who intended to either fully or partially breastfeed were able to give their baby the opportunity to suckle within an hour of the birth (99/192 or 52%) (Table 1). For 19% of the women, the opportunity was delayed for more than five hours and this affected twin pregnancies (70%) more than singletons (16%). Although not statistically significant, there was a trend towards increasing delays in the first opportunity to breastfeed as estimated blood loss increased (Trend Chi2, p = 0.13) (Figure 1). Women who were able to give their baby the opportunity to suckle within two hours of birth were more likely to fully breastfeed at baseline (Trend Chi2, p < 0.01) (Figure 2).

**Table 1:** Timing of first opportunity to suckle for babies of mothers who intended to breastfeed.

	Singletons	Twin babies
Time since birth	n = 182	n = 20*
30 minutes or less	57 (31.3%)	5 (25%)
31 minutes to 1 hour	39 (21.4%)	-
Up to 2 hours	30 (16.5%)	-
Up to 3 hours	10 (5.5%)	-
Up to 4 hours	10 (5.5%)	1 (5%)
Up to 5 hours	7 (3.9%)	-
More than 5 hours**	29 (15.9%)	14 (70%)

* Includes one mother twin pair where mother indicated she intended to formula feed but said babies were given opportunity to breastfeed

**Responses ranged widely from 8 hours to 50 hours.

**Figure 3:**
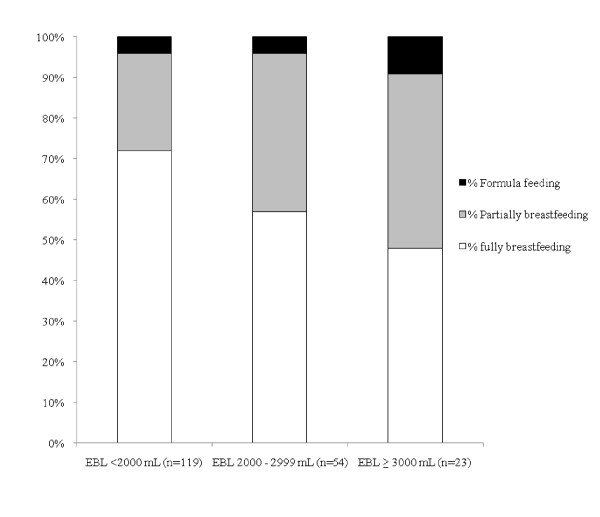
**Breastfeeding status in first postpartum week among mothers with significant PPH who intended to breastfeed, by estimated blood loss**.

Among women intending to breastfeed, those with the higher estimated blood loss were less likely to fully breastfeed in the first postpartum week than women with lower estimated blood loss (Figure 3). In summary, just over 70% women with PPH of < 2000 mL were fully breastfeeding in the first postpartum week, whereas less than 50% of those with blood loss ≥ 3000 mL were able to do so (Trend Chi2 p = .01). In crude analyses, there was a marginally significant association between our a priori exposure of interest (estimated blood loss) and the likelihood of partially breastfeeding or bottle/formula feeding (Table 2). Method of birth and timing of the first opportunity to suckle were co-linear and each of these exposures was significantly associated with the likelihood of partially breastfeeding or bottle/formula feeding. Adjusted ORs are also shown in Table 2 for our a priori exposure of interest adjusted for each of these exposures separately.

**Table 2:** Predictors of not fully breastfeeding in the first postpartum week (n = 187).

	Partial or no breastfeeding (compared with full breastfeeding)		
	**Unadjusted OR**	Adjusted OR*	Adjusted OR**
	**(95% CI)**	**(95% CI)**	**(95% CI)**

**Estimated blood loss**			
< 2000 mL	1 (referent group)	1 (referent group)	1 (referent group)
2000-2999 mL	1.91 (0.96, 3.78)	2.05 (0.99, 4.22)	1.76 (0.87, 3.58)
≥ 3000 mL	2.51 (0.96, 6.60)	2.46 (0.90, 6.72)	2.03 (0.74, 5.65)
**Method of birth**			
Vaginal	1 (referent group)		
Assisted vaginal	5.45 (2.19, 13.59)		
Caesarean section	2.32 (1.16, 4.64)		
**Time to first suckle**			
≤ 1 hour	1 (referent group)		
≤ 2 hours	1.2 (0.48, 2.97)		
≤ 5 hours	1.93 (0.79, 4.70)		
> 5 hours	3.73 (1.66, 8.40)		

* Adjusted for method of birth

** Adjusted for time to first opportunity to suckle

Overall, just over half of the participants (53%) received a transfusion of blood or blood products in the first postpartum week. Rates were highest among women with estimated blood loss ≥ 3000 mL (96%) where all but one woman were transfused (one refused transfusion being a Jehovah's Witness). In contrast, 37% of those with estimated blood loss < 2000 mL and 70% with estimated blood loss 2000 -- 2999 mL received a transfusion of blood or blood products in the first postpartum week. Overall, among women intending to breastfeed, there was no statistically significant difference in full breastfeeding rates in the first postpartum week by transfusion status (61% among women with transfusion versus 70% among women without transfusion). This was true within each estimated blood loss category (< 2000 mL: 73% versus 71%; 2000 -- 2999 mL: 53% versus 69%; ≥ 3000 mL: 50% for the 22 women transfused, only 1 woman not transfused and she partially breastfed).

### Duration of breastfeeding

**Figure 4:**
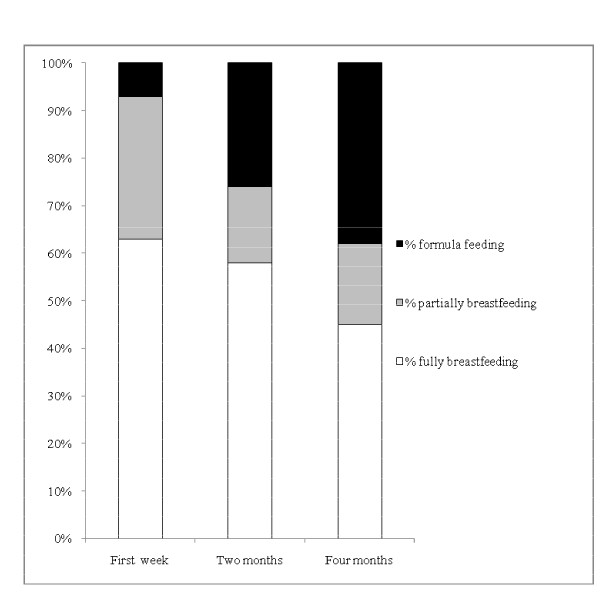
**Breastfeeding status in first postpartum week, at two and at four months postpartum among women with significant PPH**.

**Figure 5:**
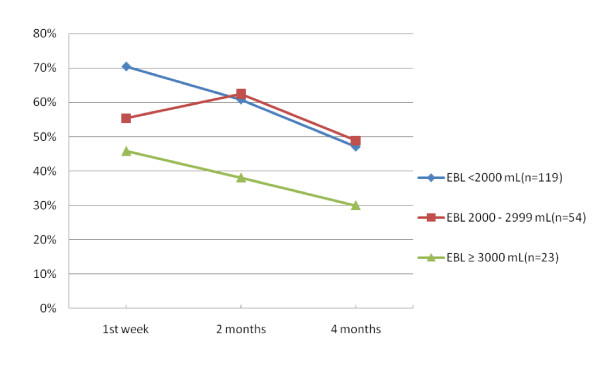
**Percentage of women with significant PPH fully breastfeeding in the first postpartum week and at 2 and 4 months postpartum, by estimated blood loss**.

Overall, 63% of women with significant PPH were fully breastfeeding in the first postpartum week, 58% at two and 45% at four months postpartum (Figure 4). Note that for all mothers of twins, the method of feeding for each twin of each pair was the same at all time points. Stratifying the data by estimated blood loss, rates of full breastfeeding were lowest at all time points for women with the highest estimated blood loss (Figure 5). There was some recovery in terms of reversion from partial to full breastfeeding by two months postpartum among women in the intermediate category (2000-2999 mL), but not among those with greater estimated blood loss.

### Maternal perception of baby's health

At two months postpartum, 129 (75%) participants rated their infant's health as excellent, 37 (22%) as good, with only 4 (2%) rating infant health as fair and 1 (0.6%) as poor. Corresponding figures at four months were 75%, 20% 4% and 1% respectively.

### Mastitis

Overall, at two months 37/170 (22%) participants reported that a breast infection or mastitis had been either a minor or major problem for them in the preceding two months and at four months the proportion was 22/162 (14%). No association was found between estimated blood loss and risk of problems with mastitis.

### Analysis of women's written comments relating to breastfeeding

Thirty nine women included references to their breastfeeding experiences in their written comments in the questionnaires. Three major themes were identified: 1) Difficulty initiating or sustaining breastfeeding, 2) Need for education and support; and 3) Emotional sequelae.

#### Difficulty initiating or sustaining breastfeeding

Women wrote of many factors that made initiating breastfeeding difficult. These included physical separation from the baby, fatigue, mobility and posture problems, and delayed milk production. One woman summed up her experience as:

*"Breastfeeding was much harder than I thought and my delivery experience made it harder." *(ID 52, primipara, 1500 mL blood loss, 2 months postpartum).

One woman described her main concerns after the birth as:

*"Separation from baby one and a half hours after birth and for about three plus hours whilst in theatre. Delay with getting breastfeeding underway." *(ID 125, primipara, 1500 mL blood loss, first week postpartum).

Women also wrote of insfficient and slow production of colostrum/milk as well as sheer inability to breastfeed "....*due to no energy because of the PPH."*

Reasons cited for ongoing breastfeeding difficulties or cessation included: complications such as mastitis and nipple pain, stress and inconvenience - particularly when the breast milk was expressed, and meeting the physical demands of breastfeeding while recovering from the PPH. Having mastitis or a breast abscess was reported variously as a reason for readmission to hospital, ongoing physical concerns and a cause of discomfort. In general, breast discomfort was a commonly cited problem along with "*nipple damage and soreness*" and "*sore breasts"*. One woman began supplementing with formula as she was suffering "*deep pain in the breasts following a feed"*' and tender breasts. Other discomfort suffered included postural problems and "*stiff/sore neck" *due to tenseness during breastfeeding. With regard to the physical demands of both breastfeeding and recovering from the PPH, one participant wrote:

*"I feel that most of my problems health wise are due to breastfeeding and the effort that it takes both in time.........and in physical effort (strain on body). However I think the reality of my having the after birth bleeding and losing so much blood was just that it took so much longer than normal (other mothers) to feel like I was normal." *(ID 387, primipara, 1800 mL blood loss, 4 months postpartum).

Any additional health problems exacerbated the problem:

*"Not only did I lose a lot of blood with my haemorrhage, but I also got pneumonia in my left lung so I couldn't do much. Even breastfeeding my baby I became breathless." *(ID 363, primipara, 2500 mL blood loss, 2 months postpartum).

#### Need for education and support

Women's written comments reinforced the importance of appropriate and timely support and advice for successful breastfeeding. Women wrote mainly of their experiences with midwives; women who had a positive experience used words such as "*help*" and "*information"*. One participant said that the midwife *"ensured the baby was put to the breast as soon as possible"*. This was found to be "*reassuring"*. Conversely, women who had negative experiences used words such as "*unhelpful"*, citing reasons such as getting "*mixed opinions*" and "*conflicting advice". *For example, one woman wrote in relation to attachment problems:

*"The midwives weren't very good with me trying to breastfeed. I had attachment problems and they made it worse. I had to consult a lactation specialist to help me with this after I left hospital as I got too much conflicting advice at the hospital." *(ID 171, primipara, 2500 mL blood loss, 2 months postpartum).

Not all advice given by healthcare providers was deemed helpful or was followed. One participant said she chose not to continue to take the analgesia she was prescribed. She said that as a result she *"felt absolutely fantastic" *and "*breastfeeding improved as the baby wasn't as drowsy."*

Issues of timely access to appropriate services were also raised. One participant wrote:

*"I would have found it helpful to have spent some time at the breastfeeding clinic or have been encouraged to do so." *(ID 52, primipara, 1500 mL blood loss, 2 months postpartum).

One woman consulted a lactation specialist independently when she left hospital which she found helpful and another regretted that she was not seen by a midwife at home:

*"...would have been good to have had more assistance after left hospital with breastfeeding (access to lactation consultants) as milk didn't come in until last day." *(ID 213, primipara, 1500 mL blood loss, 2 months postpartum).

In relation to the impact of the PPH, one participant noted:

"*Nobody said the bleed could affect my milk supply/baby's weight." *(ID 320, primipara, 2000 mL blood loss, 2 months postpartum).

Another, although advised that her milk may be delayed, felt that not enough consideration was given to her *"hungry" *baby's feeding needs and recommended:

*"...... that mothers suffering PPH be advised of its potential impact on milk supply and be offered feeding support earlier rather than later." *(ID 319, primipara, 2000 mL blood loss, first week postpartum).

Some things were mentioned which might have made a difference. For example, in one woman's words:

*"...now it's bothering me that the doctors in hospital would just not listen to me about getting a blood transfusion earlier (I had a PPH previously with my first child) and my recovery and difficulties with breast feeding would have been so much better!" *(ID 393, multipara, 1800 mL blood loss, 4 months postpartum).

And another:

*"I wish they had given her some formula just for a couple of days. I went to *(residential mother-baby unit)* and they encouraged very vigilant intense breastfeeding i.e. very regular and both breasts X2 each time -- I just wish the hospital staff had done that from the start." *(ID 320, primipara, 2000 mL blood loss, 2 months postpartum).

#### Emotional sequelae

A number of women poignantly expressed the emotional consequences of their inability to realise their goal of breastfeeding their babies. Some wrote of the stress associated with breastfeeding. The stressors included expressing and supplementary feeding, as well as breastfeeding twins. One mother spoke of "*confusion and frustration" *associated with conflicting advice from postnatal midwives and another had *"concerns about whether I can produce enough milk." *Some women found breastfeeding difficult, especially when they had to express breast milk, which was referred to as *"inconvenient" *by one woman who persevered for four months and "*tiring" *by another. Disappointment and regret was mentioned by mothers who had been unable to feed. The intensity of this feeling varied; one mother was resigned:

"*I do sometimes wish I could have continued breastfeeding, but it just didn't work out*." (ID 158, primipara, 3000 mL blood loss, 4 months postpartum).

Another was devastated and attributed her negative experience to the PPH:

"*My dream of breastfeeding has been shattered due to no energy because of the PPH so have started feeling quite down." *(ID 393, multipara, 1800 mL blood loss, 4 months postpartum).

Yet another expressed her disappointment thus:

"*I feel disappointed as due to my blood loss I have not been able (or my baby) to extract enough colostrum and it has been coming in slowly as I have not had the chance to get enough rest. The outcome has been a jaundiced baby placed in an infant incubator, and having to be given formula to supplement her needs, which is something I really did not want to do." *(ID 225, primipara, 3000 mL blood loss, first week postpartum).

One woman expressed feelings of uselessness and the onset of symptoms of depression to her failure to breastfeed:

"... ...*not being 'mobile' meant the only thing I can do for my new son was to breastfeed, and when that fell over, upset and feelings of uselessness set in....I did begin to develop postnatal depression when I was unable to continue breastfeeding which I found very difficult and disappointing*." (ID 393, multipara, 1800 mL blood loss, 4 months postpartum).

## Discussion

This study reports, for the first time, breastfeeding outcomes in a cohort of women experiencing a PPH. Despite experiencing a significant, and in some cases life threatening blood loss, participants in this study achieved remarkably good rates of both initiation and duration of full breastfeeding. In the first postpartum week, 63% were fully breastfeeding their babies and by four months postpartum 45% were still doing so. Further, maternal ratings of infant health at both two and four months were high, with over 95% rating their infant's health as excellent or good at both time points.

The population from which our sample was drawn has a high rate of initiation of full breastfeeding and indeed over 85% of the women in our sample expected to fully breastfeed their babies from birth. While in this cohort only 63% were able to achieve this goal, in general, women may not always achieve their desired goals in relation to breastfeeding. For example, in a UK study only 75% of women expressing a desire to breastfeed actually initiated breastfeeding after delivery [24] and in a population of women in the United States, 61% expected to exclusively breastfeed whereas 51% actually did so [21]. Numerous factors may impact on the ability to successfully establish full breastfeeding despite antenatal intentions. For example, women who have caesarean deliveries or other interventions such as epidural anaesthesia, as well as those whose infants are admitted to a special care nursery, are less likely to be fully breastfeeding at discharge [17,25,26]. Fetal stress, birth trauma and maternal stress are also known to contribute to delayed onset of copious milk secretion [12,27]. Thus, for women experiencing a PPH there is the potential for multiple factors/pathways to impact on breastfeeding success: vascular insult to the pituitary gland as a direct consequence of the PPH (in the most extreme cases, Sheehan's syndrome); interventions and stress during labour and delivery; delay in breastfeeding initiation due to separation from the infant; and, further, maternal exhaustion may result in the infant receiving early complementary feeds and thereby subsequent difficulties for the mother in establishing full breastfeeding.

In our cohort of women with significant PPH we have documented delays in breastfeeding initiation. Just under half of the mothers who intended to breastfeed were unable to initiate breastfeeding within the recommended time period of one hour after birth, and delays in initiation were more common among women with higher estimated postpartum blood loss. Delayed initiation of breastfeeding is understandable in the context of a high proportion of mothers possibly requiring transfer to theatre, care in a HDU or ICU, or of their babies requiring special care. Among women for whom initiation of breastfeeding was delayed, we found lower rates of full breastfeeding -- or, conversely, higher rates of partial breastfeeding and of formula feeding. This is of concern, as it is known from other studies that women who commence partial, rather than full breastfeeding, are at risk of early cessation of breastfeeding [17]. In our cohort, partial breastfeeding in the first postpartum week is likely to be indicative of problems in establishing breastfeeding, including delayed lactogenesis Stage II.

We were also able to examine the association between severity of blood loss and breastfeeding in the first postpartum week. We found that estimated blood loss was negatively associated with full breastfeeding in the first postpartum week (p for trend 0.01). After adjustment for method of birth and timing of the first opportunity to suckle, this association almost reached statistical significance using p value of ≤ 0.05. Further exploration of this association in a larger study is warranted.

We also report for the first time data on duration of breastfeeding to four months postpartum among women experiencing a PPH. Many factors are known to influence the duration of breastfeeding [28] and this may include experiencing a traumatic stressor, such as a PPH [29]. In our cohort of women with significant PPH, full breastfeeding rates at two months postpartum and at four months postpartum were 58% and 45% respectively. Rates of full breastfeeding in the first postpartum week and at both two and four months postpartum were lowest among the subgroup of women with the highest estimated postpartum blood loss. Overall, for women with significant PPH, the rates of full breastfeeding fall well short of the WHO recommendation of 100% full breastfeeding for six months, although current data indicate that this is rarely achieved in any populations [21,30,31]. The rates observed do however compare quite favourably with other published full breastfeeding rates for general populations of Australian women (87% in first week and 57% at four months) [17] and are substantially higher than, for example, US general population data (51% at one week and 22% at three months) [21]. Our observation that among women with 2000-2999 mL estimated blood loss there was some recovery in terms of reversion from partial to full breastfeeding by two months postpartum, is encouraging. This suggests that that even if full breastfeeding cannot be established immediately, there is the prospect of doing so later, and offers potential for interventions to support and encourage women to continue breastfeeding following a significant PPH despite early difficulties.

This study was not designed to examine a possible effect of receiving a transfusion of blood or blood products on breastfeeding success. Overall, lower rates of full breastfeeding were actually observed among women who received transfusion compared with those who did not. This is most likely a reflection of the fact that women receiving a transfusion were less well than those who did not, thereby confounding any association between transfusion and initiation of full breastfeeding.

Consistent with the quantitative results, our qualitative data indicate that difficulties with breastfeeding may be due to delayed lactogenesis Stage II in this population, with women reporting delays in onset of milk secretion. In addition, early separation from their baby, their stressful birth experience, ongoing fatigue and the physical sequelae of PPH were all cited by women as factors influencing their ability to successfully breastfeed. The qualitative data are also consistent with the concept that inability to successfully breastfeed is not benign and has emotional sequelae including disappointment, loss, regret and sense of failure [13]. Women's accounts of their breastfeeding experiences also highlight the crucial role of health care providers in supporting women to breastfeed, in particular, providing them with adequate information, reassurance and practical advice.

### Study limitations

This is a descriptive study and as such subject to a number of limitations. Firstly, it was not designed to establish causal associations between the PPH and breastfeeding outcomes. Also, while clinically significant Sheehan syndrome is now very uncommon [8], we did not assess signs of pituitary failure which might contribute to maternal lactation failure. Nonetheless, in the absence of other studies to date we are able to provide descriptive outcome data which may prompt further studies to examine specific associations and causal pathways. Secondly, the information regarding prenatal intention to breastfeed is subject to recall bias as it was asked in the postnatal period -- albeit in the week immediately postpartum. Thirdly, our outcome data are limited to four months postpartum and it would be important in future studies to extend the follow-up period to at least six months -- the recommended duration of full breastfeeding. In addition, it would be valuable to include more detailed assessment of maternal and infant experiences of breastfeeding and include objective measurements of milk production, proportion of daily fluid intake from breast milk and of infant growth patterns in the first few days and then over time. Fourthly, there are potential issues in relation to selection of our sample. It is possible that we may have recruited a 'healthy' cohort with possible bias towards women who were less severely affected by their birth experiences. Further, recruitment took place across 17 different sites and while ideally we would have recruited in direct proportion to the number of women in each site meeting the eligibility criteria, in fact there was some bias towards those hospitals able to commit time and resources to the study. Lastly, we cannot ignore a possible Hawthorne effect, whereby practices within participating hospitals may have altered as a result of being under study, possibly in the direction of providing greater support and attention to women experiencing a PPH perhaps thereby positively influencing breastfeeding rates.

In relation to women's written accounts of their breastfeeding experiences, we did not specifically set out to examine this in-depth, rather, their comments are serendipitous. Nonetheless, in the absence of other published information we consider them worthy of reporting, with the acknowledgment that of the 206 participants only 39 wrote about breastfeeding-related issues and that responses are most likely from women who had problems or negative experiences. Future qualitative studies might explore breastfeeding experiences in greater depth and focus more specifically on those factors associated with both negative and positive breastfeeding experiences among women following a PPH.

## Conclusions

The observations reported here support the hypothesis that following a significant PPH, women are able to successfully breastfeed, but may find it difficult for various reasons. Women with greater blood loss are more likely to be adversely affected and this may be related, in part, to delays in initial contact with their baby as a consequence of the PPH. These findings have implications for postnatal care, as these women may require greater support, education and assistance in initiating and sustaining breastfeeding. In particular, enabling the opportunity for the newborn to suckle as soon as is practicable should be encouraged.

Maternity services should identify and address modifiable factors that hinder the ability of women with PPH to successfully initiate and sustain full breastfeeding and recognise and treat delayed or failed lactogenesis Stage II where necessary [32].

## Competing interests

The authors declare that they have no competing interests.

## Authors' contributions

JT, DE and CR conceived the study design. JT acquired the data, carried out data analysis and revised the manuscript. LJH carried out qualitative data analysis and wrote the first draft of the manuscript. CR contributed to data analysis and interpretation and critically reviewed subsequent drafts of the manuscript. DE had the original idea for the study, obtained funding, contributed to interpretation of the data and critically reviewed the draft manuscripts. All authors read and reviewed the final manuscript.
